# Renaissance of Cardiac Imaging to Assist Percutaneous Interventions in Congenital Heart Diseases:The Role of Three-Dimensional Echocardiography and Multimodality Imaging

**DOI:** 10.3389/fped.2022.894472

**Published:** 2022-05-19

**Authors:** Martina Avesani, Sok-Leng Kang, Zakaria Jalal, Jean-Benoit Thambo, Xavier Iriart

**Affiliations:** ^1^Department of Pediatric and Congenital Cardiology, M3C National Reference Centre, Bordeaux University Hospital, Bordeaux, France; ^2^Department of Pediatric Cardiology, Alder Hey Children’s Hospital, Liverpool, United Kingdom; ^3^Institut Hospitalo-Universitaire (IHU) Liryc, Electrophysiology and Heart Modeling Institute, Bordeaux University Foundation, Pessac, France

**Keywords:** interventional echocardiography, 3D echocardiography, multimodality imaging, transcatheter procedures, advanced imaging

## Abstract

Percutaneous interventions have completely refashioned the management of children with congenital heart diseases (CHD) and the use of non-invasive imaging has become the gold standard to plan and guide these procedures in the modern era. We are now facing a dual challenge to improve the standard of care in low-risk patients, and to shift our strategies from the classic open chest surgery to imaging-guided percutaneous interventions in high-risk patients. Such rapid evolution of ultrasound technologies over the last 20 years have permitted the integration of transthoracic, transesophageal and intracardiac echocardiography into the interventional workflow to improve image guidance and reduce radiation burden from fluoroscopy and angiography. Specifically, miniaturization of transesophageal probe and advances in three-dimensional (3D) imaging techniques have enabled real-time 3D image guidance during complex interventional procedure, In addition, multimodality and fusion imaging techniques harness the strengths of different modalities to enhance understanding of anatomical and spatial relationship between different structures, improving communication and coordination between interventionalists and imaging specialists. In this review, we aim to provide an overview of 3D imaging modalities and multimodal fusion in procedural planning and live guidance of percutaneous interventions. At the present times, 3D imaging can no longer be considered a luxury but a routine clinical tool to improve procedural success and patient outcomes.

## Introduction

Percutaneous interventions have completely refashioned the management of children with congenital heart diseases (CHD) and the use of non-invasive imaging has become the gold standard to plan and guide these procedures in the modern era.

Transesophageal probe miniaturization and advanced live Three-dimensional (3D) imaging have permitted a massive step forward in the ultrasound imaging guidance, and 3D echocardiography is now recommended to guide catheter-based interventions (1), since it provides high-quality and real-time images without additional X-rays exposure.

In addition, fusion imaging systems, anatomical intelligence and multimodality imaging can improve the understanding of anatomical and spatial relationship between different structures and provide better communication and coordination between interventionalists and imaging specialists.

In this review, we aim to provide a “state of the art” of the different imaging technologies that have been integrated to the catheterization laboratory workflow and that are going to shape the future of congenital transcatheter procedures.

## Echocardiographic Techniques

### Transthoracic Echocardiography

Transthoracic echocardiography is the milestone of imaging in CHDs and should be performed before and after any cardiac catheterization procedure. In the pre-procedural planning it guarantees a complete anatomical assessment; it can be helpful in refining the diagnosis in patients referred for percutaneous procedures, and in determining the need for mechanical support in presence of ventricular dysfunction (2). In the post-procedural time, it is fundamental to rule out complications such as pericardial effusion, device dislodgment, valve or ventricular function impairment.

Although the role of TTE has been limited in the catheterization laboratory in favor of transesophageal and intracardiac echocardiography in older patients, it still plays a key role in some procedures, such as patent ductus arteriosus closure in premature babies (3), atrial septostomy (4) and aortic and pulmonary valvuloplasty in children (5, 6).

### Transesophageal Echocardiography

#### General Considerations

Transesophageal echocardiography is considered the gold standard technique to guide interventional procedures. Miniaturization of probes allows the utilization in neonates, with a suggested weight-limit of > 3.0–3.5 kg for the mini-multiplane probe and > 2.5 kg for the micro-multiplane one (7). Adult probes can be safely used in children weighing at least 18–20 kg (8) to take advantage of the 3D technology, nowadays available only in adult probes, but miniaturization of 3D TEE probes in a close future will allow another step forward in advanced 3D guiding for percutaneous interventions.

Transesophageal Echocardiography (TEE) image quality is better than TTE when analyzing atrial and ventricular defects, left sided lesions and to monitor the catheter position (9), due to the esophageal position of the ultrasonic transducer and to the possibility of manipulating the probe in in several directions. It can be advanced and withdrawn through the esophagus (from upper esophageal to deep transgastric views) allowing the visualization of more inferior and superior cardiac structures, respectively, while a clockwise and counterclockwise rotation permits to assess right and left side structures. Also, the probe tip can be gently anteflexed or retroflexed to better visualize anterior and posterior structures and flexed to the left or to the right; this left-to-right flexion is usually not available in pediatric probes, due to the small dimension of the esophagus. Lastly, the multiplane probe allows a rotation of the scanning plane through 180° (7).

Transesophageal Echocardiography (TEE) decreases fluoroscopic time, radiation (10) and contrast load, and this is very important considering that pediatric congenital patients will undergo several catheterizations during their life.

For patient comfort, TEE in the catheterization laboratory is usually performed under sedation/general anesthesia, thus requiring orotracheal intubation. Probe insertion requires expertise to avoid esophageal injuries. Imagers should also be familiar with some technical features of probes, such as the critical tip temperature above which probes are shutdown to prevent esophageal burns (usually set at 42.0°C) (11).

#### 2D or 3D Transesophageal Echocardiography? Both!

Echocardiography is nowadays such a multimodality tool that imagers can easily pass from bidimensional echocardiography (2D) standard images to 3D detailed anatomy of cardiac structures and multiplane reconstruction.

3DE adds value to 2D technique, since it allows a real 3D visualization of complex cardiac anatomies that is, in 2DE, only in imagers’ mind. However, it should be remembered that the acquisition of good 2D images is fundamental to obtain valuable 3D data. Thus, 2D and 3D echocardiography (2DE, 3DE) have a complementary role during interventional procedures, and it is recommended to include 2D, Color flow and spectral Doppler imaging as part of baseline TEE assessment (7).

Currently available 3D TEE transducers operating frequencies ranges between 5 and 7 MHZ. There are different 3D acquisition modes that can be used during procedures guidance, including (1, 12):

- 2D simultaneous multiplane: it gathers 360° images, so more than one plane of visualization is available simultaneously (X-plane), with the main disadvantage of a low temporary resolution.
- Real-time 3D (live 3D and 3D zoom): it allows an adjustable pyramidal volume acquisition (around 30° × 60°). It requires a small region of interest to preserve a satisfactory spatial and temporal resolution (frame rate: 20–30 HZ) (13). It is the most used modality in interventional echocardiography, to assess atrial and ventricular septal defects (ASD, VSD) and to guide their percutaneous closure (14).
- ECG-gated multi-beat: the acquisition of a large field of view (usually 60° × 60°) with high-temporal resolution (frame rate: > 30 HZ) makes this modality widely used in pediatric interventions. The drawback related to the potential creation of breathing artifacts (stitching artifacts), can be easily resolved during interventional echocardiography by briefly suspending mechanical ventilation.
- 3DE color flow Doppler: it is an additional tool to the above modalities, but its use reduces temporary resolution.

### Intracardiac Echocardiography

Phased-array intracardiac Echocardiography (ICE) requires an additional venous femoral approach (8 or 10 F) to advance the ICE catheter to the right atrium and displays cardiac images from inside the heart. The transducer can be deflected in four directions and is available with 2D and Color-Doppler techniques, but imaging is limited to a single longitudinal plane (90°) (7). It provides a real-time assessment of cardiac anatomy during interventional procedures such as ASD closure, and a good guidance for catheter manipulation in relations to the different anatomic structures (15).

3D technology is also available, with the possibility of acquiring a volume of 60° × 15° at a volume rate of 20, on a 10-French probe (16, 17). However, the experiences are limited and the costs still high.

Intracardiac Echocardiography (ICE) can be performed by the interventionalists themselves, usually under local anesthesia, thus avoiding intubation and the risks related to esophageal trauma. It also reduces fluoroscopy exposure and procedural timing (18). However, a learning curve is necessary to understand how to manipulate the probe (19) and it should not be considered a risk-free technique, since cardiac and venous perforation, arrhythmias, and thromboembolism have been described (9).

### May I Say?

To provide an efficient procedure and the best as possible result, a good communication and coordination between interventionalists and imaging specialists is mandatory, starting from pre-procedural planning to post-procedural monitoring. During the procedures, they should use a common terminology and spatial orientation. The echocardiographer must be familiar with procedures and guarantee the best echocardiographic projections to help the interventionalists who, in turns, should communicate effectively with the imagers and pay attention to his suggestions (20).

These considerations lead to an important message: in the Cath Lab, physicians cannot improvise as imagers. As consequence, a dedicated and standardized training in large-volume centers (> 500 TEE/year) under the guidance of senior experts, and including a defined number of different procedures as well as the possibilities of obtaining international certifications is advocated and should be followed by cardiologists who will be in charge of guiding percutaneous procedures, as recommended by scientific societies (21). This is even truer in the context of CHDs procedures, where the variety of anatomic substrates, lesions and surgical results should be interpreted and managed by trained operators.

## Echo-Guidance in the Cath-Lab: Clinical Applications

### Ostium Secundum Atrial Septal Defect Closure

Percutaneous technique is now considered the treatment of choice for OS-ASD closure (22). TTE is usually sufficient in the preoperative phase to select patients by evaluating rims and size of defects. However, TEE is the best imaging technique for carefully assess the interatrial septum. Periprocedural imaging should assess:

- number, size, and area of defects: for a single defect, the largest diameter measured in two perpendicular planes is taken for sizing. Defects > 38 mm are considered as complex, but percutaneous closure has been reported in highly expertise centers (23).
- rims’ size: for a complete 2D evaluation, a rotation from 0° to 180° should be performed; rims are measured at approximately 0° (posterior and antero-inferior), 45° (posterior-inferior and antero-superior), 90° (posterior-inferior and posterior-superior), 135°(coronary-sinus and posterior-superior) (24); to date, percutaneous closure of ASD with deficient posterior-inferior rim (< 5mm) is not recommended (25) due to the risk of device embolization and potential right-left shunt caused by straddling of the inferior vena cava. Deficiency in other rims is not a contraindication to percutaneous closure.
- features of the interatrial septum, particularly the presence of atrial septal aneurysm and/or multifenestrated septum (26).
- pulmonary venous returns.

3DE should routinely be used in ASD assessment, and some live 3D modalities can rapidly be acquired during procedures.

While X-plane allows the simultaneous visualization of two perpendicular planes, thus granting rims and diameters assessment, 3D zoom from the mid—esophagus position provides *en face* views of defects, either from the right or from the left atrium, and a more precise localization and definition of the surrounding structures, intuitive for interventionalists (27, 28). Also, it estimates more accurately the shape (oval or round), the maximum and minimum diameter and area of defect, thus allowing a correct device choice without the need for balloon sizing (29).

**FIGURE 1:**
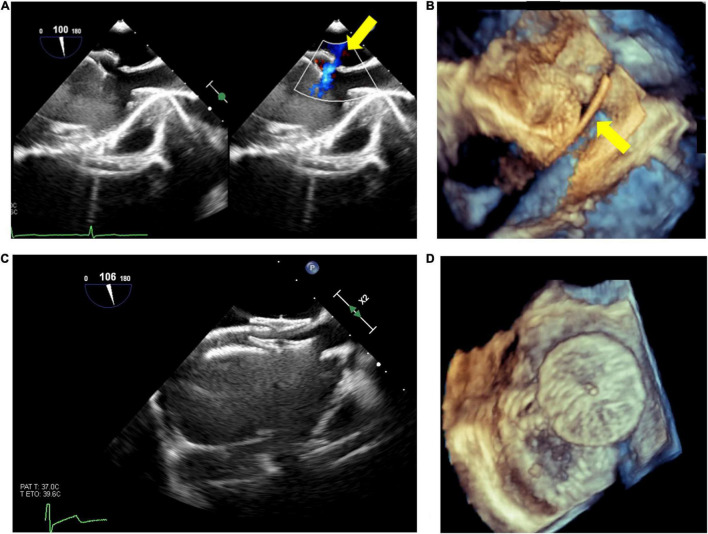
Atrial septal defect (ASD) closure with two devices. Residual ASD (yellow arrow) after device release **(A)**; live 3D echo showing the guidewire passage (yellow arrow) through the residual defect **(B)**; 2D visualization of the devices **(C)**; 3D visualization of devices position and shape **(D)**.

Live 3D is also particularly helpful in complex cases, such as multiple/fenestrated defects or when multiple devices are needed (30). In case of multiple defects guarantees a better visualization of both catheters position and defects, facilitating catheter crossing the septum through the main defect to assure the correct positioning of the main device. After device release, 3D imaging provides detailed information about device position, shape and stability, residual shunts, and potential interference with aortic/atrio-ventricular valves or with systemic/pulmonary venous return (31). An example of double device implantation for residual ASD is shown in Figure 1.

### Ventricular Septal Defects Closure

Transcatheter closure of muscular and, to a lesser extent, perimembranous ventricular septal defects (mVSD, pmVSD) is nowadays a valid alternative to surgical closure (32).

Imagers should know the main steps of the procedure, which could be performed with either an anterograde or retrograde approach.

Transthoracic Echocardiography (TTE) and TEE, either 2D or 3D, are mandatory in the preoperative and intraoperative phase respectively, to determinate the VSD location, morphology, size, the relationship to the aorta (for pmVSD) and to make the device selection choice.

Real-time 3DE allows a unique *en face* view of VSDs from the right or left ventricular side and a complete evaluation of relative changes in VSD area throughout the cardiac cycle, with an overall acquisition time close to that of a 2DE study (33). Then, it better defines perimembranous ventricular septal aneurysms, especially regarding the presence and extension of accessory tricuspid tissue (34); it permits to visualize the tricuspid valve chords and the defect in a single projection as well as to exclude the presence of aortic valve prolapse, which can be difficult to visualize on 2DE (35).

As for pmVSDs, the choice of the device size is made considering that the device left-side size should be at least twice the minimum VSD diameter on the right ventricular septum and equal to or 1–2 mm greater than the diameter of the VSD at the left ventricular opening (36). As for mVSD, an occlude 1–2 mm larger than the maximum size of the defect is chosen (37).

**FIGURE 2:**
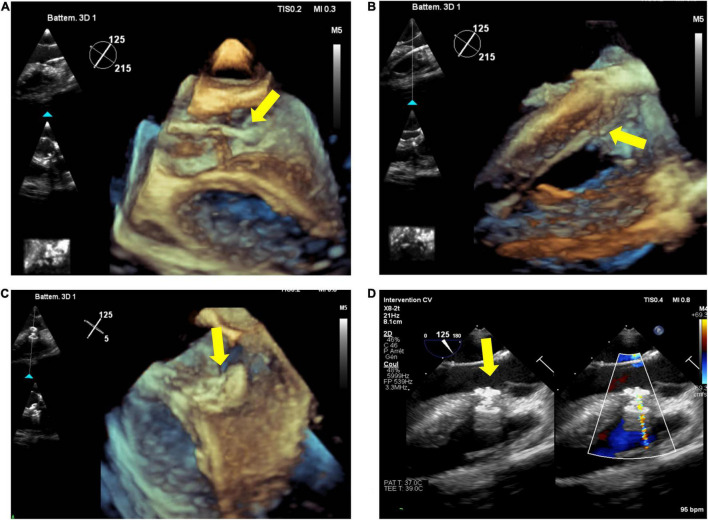
Percutaneous closure of perimembranous ventricular septal defect (pmVSD). Transesophageal echocardiography (TEE), especially 3D, permits visualization of catheter passage through the aortic valve **(A)** and in the right ventricle **(B)** (yellow arrow), and live monitoring during **(C)** and after device release **(D)** (yellow arrow).

Live 3DE is also helpful during and after device deployment, to check the position of the two discs across the interventricular septum, and to look out for potential residual shunt as well as for aortic and tricuspid valve function. An example of perimembranous VSD closure is shown in Figure 2.

### Coronary Artery Fistulae

Transcatheter closure of coronary artery fistulae is feasible, but it requires an adequate preoperative assessment, including TTE and, sometimes, cardiac Computed-Tomography.

Echocardiographic imaging of fistulae can be challenging; 2D TTE and 2D TEE can give information about origin and distal exit points of fistula, as well as volume overload and severity of shunt (38), but they may have limitations in tracing its course and precisely define the site of drainage (39). Although limited, some experiences of percutaneous procedures under 3D TEE have been reported, highlighting how 3DE can add additional values in these complex scenarios (40, 41).

### Percutaneous Interventions in Patients With Fontan Circulation and Atrial Switch

Few data is available on the role of echocardiography during percutaneous interventions on patients with Fontan circulation. 3D TEE is useful to close fenestration situated in hardly approachable positions (42) and to identify additional leaks in the circuit as well as the presence of thrombus during electrophysiologic procedures (43).

**FIGURE 3:**
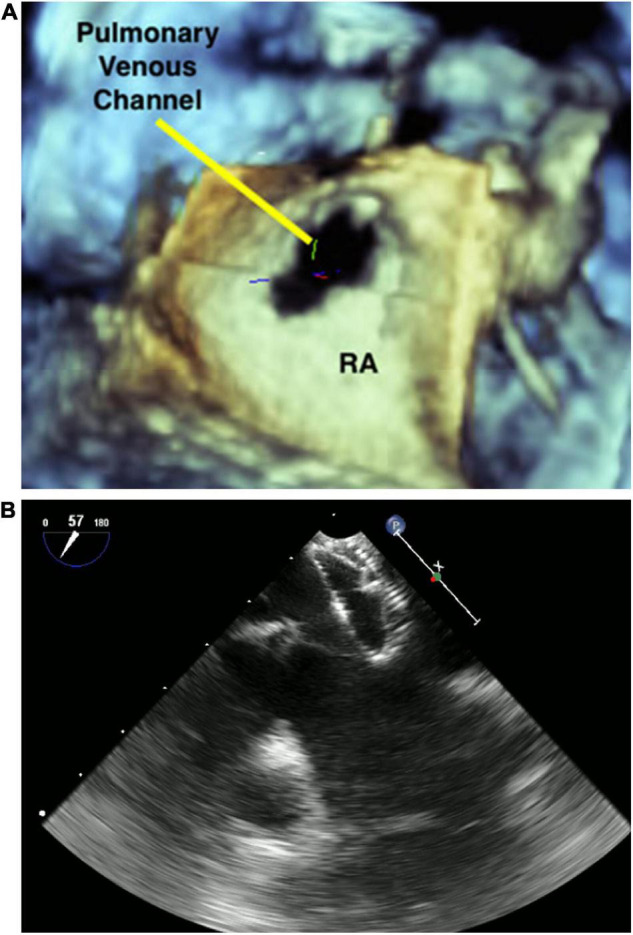
3D image of pulmonary venous channel in a patient who underwent atrial switch **(A)**. Baffle leak closure **(B)**.

Among the long-term sequelae after atrial switch operation for D-Transposition of Great Arteries, baffle leaks and obstruction are common. Although the use of 2D TEE to aid stent placement for systemic venous baffle obstruction/baffle leak is known to be safe and effective (44) only 3DE can precisely localize the position of the baffle leak, thus allowing a successful percutaneous closure (45). An example of baffle leak closure is shown in Figure 3.

Lastly, patients with Fontan circulation or atrial switch are at-high risk for arrythmias, and catheter ablation is more and more performed. However, considering their anatomical complexity and the need for transbaffle or transconduit puncture to access the right atrium, a meticulous procedural planification is required. Puncture simulation based on computed tomography (CT) dataset is helpful in targeting the optimal puncture site, and intraoprocedural TEE or ICE are mandatory to guide the puncture and prevent complications (46, 47).

### Tricuspid and Mitral Valve Interventions

Patients with CHDs are at risk for surgical reoperations and carry a high surgical risk, often due to previous multiple interventions. So, the interest in percutaneous valve-in-valve implantations is growing, especially in patients with Ebstein anomaly with failing bioprosthesis (48) and in adults with left-sided degenerated bioprosthetic valves (49). However, promising data about transcatheter mitral valve replacement in children with CHD has also been published (50). Intraprocedural TEE facilitates valve sizing and alignment to avoid left outflow tract obstruction; it evaluates valve-in-valve function immediately after the procedure, as well as the presence of potential leaks. 3D TEE adds value in determining dysfunctional bioprosthetic valve morphology before the procedure and valve-in-valve geometry after (51).

**FIGURE 4:**
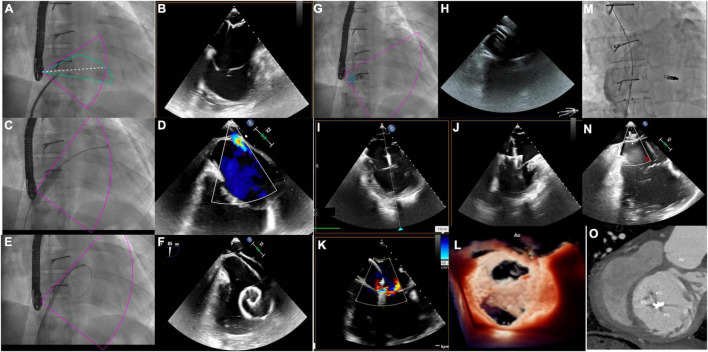
MitraClip implantation for systemic tricuspid valve regurgitation. **(A,B)** Catheter and needle are positioned at the level of the anterior part of the intra-atrial inferior vena cava channel under transesophageal echocardiography (TEE) guidance. The 80° TEE plane shows needle tenting and guarantees a distance of at least 40 mm between the tenting and the systemic atrio-ventricular valve (SAVV) and the alignment with the SAVV. **(C)** The balloon is inflated to enlarge the transbaffle access. **(D)** TEE shows the right to left shunt on color doppler after balloon inflation. **(E,F)** The guidewire is positioned in the systemic atrium to advance the MitraClip sheath. **(G,H)** MitraClip sheath is positioned on the systemic atrium at 40 mm or more from the SAVV. **(I,J)** The MitraClip system is advanced through the sheath and positioned on top of the jet origin, with the clip fully opened. Perpendicular orientation of the clip relative to the anteroseptal commissure is guided by the Xplane 3DTEE in the mid-esophagus position. **(K)** XTR MitraClip fully closed at the level of the anteroseptal commissure creates a double-orifice SAVV with two mild residual jets on color doppler. **(L)** Double-orifice SAVV on 3D Zoom true view mode after XTR clip insertion. **(M)** Inferior vena cava channel angiogram after placement of an 8 mm atrial septal defect (ASD) device to close the transbaffle access showing an absence of obstruction. **(N)** The 80° TEE view showing no residual shunt at the level of the ASD device. **(O)** Post- MitraClip computed tomography showing the position of the clip at the level of the anteroseptal commissure.

Severe systemic tricuspid valve regurgitation (STVR) is common either after atrial switch operations or in patients with congenitally corrected transposition of great arteries, and percutaneous repair with MitraClip devices might represent an option to improve symptoms in these complex patients (52, 53). To date, only one case of percutaneous treatment of STVR with MitraClip device after atrial switch has been published, and procedure summary is shown in Figure 4. Pre-procedural CT determined the appropriate baffle puncture site, while 3D TEE identified the anatomical orientation of the systemic tricuspid valve, guided real-time the puncture site, and monitored the clip release and valve function (52).

### Aortic and Pulmonary Valvuloplasty

The goal of percutaneous balloon aortic valvuloplasty in neonates and children with severe aortic stenosis is to achieve the lower as possible gradient with the least degree of aortic regurgitation.

Since oversized balloons (balloon/annular ratio > 100%) are associated with an increased risk of aortic regurgitation, precise measurements of aortic annulus are essential (54) to avoid this complication. Valvuloplasty is usually initially performed with a balloon whose diameter is 80–90% of the aortic annulus (55). 2D and 3D echocardiographic measurements are well correlated and provide accurate measurements and good procedural results (5, 56). However, 3D echocardiography assures a better understanding of the aortic annulus shape, which is usually oval, and allows more reliable values compared to 2D, which often underestimates the aortic annulus diameter (5).

Also, recent data showed that percutaneous balloon pulmonary valvuloplasty under echocardiographic guidance seems to have the same results as standard valvuloplasty but with lower contrast load and radiation, which is of great importance in pediatric patients (6).

### Patent Ductus Arteriosus Closure

Currently, PDA closure in extremely low birth weight infants relies almost exclusively on TTE guidance with high-frequency probes (57). Premature infants have tiny echocardiographic windows, thus the challenge for imagers is to take clear images moving in a very limited space.

Different measures are needed to help in device choice, such as PDA diameter at the aortic and pulmonary end, and PDA length (3). Usually, they are acquired from a high-parasternal ductal view and suprasternal views. All the measurements should be taken before instrumenting the PDA.

After PDA device deployment, the presence of residual shunt, as well as potential left pulmonary artery and aortic obstruction must be assessed. A Doppler velocity greater than 2.5 m/s associated with aliasing at Color-Doppler and 2D images consistent with obstruction should advocate, if possible, device repositioning (57).

### Fetal Cardiac Intervention

In parallel with the improvement in fetal echocardiography and prenatal diagnosis, the interest in performing percutaneous interventions in fetuses either carrying potential progressive diseases through gestation or at high risk for demise *in utero*/life threatening at birth is growing (58).

Fetal aortic stenosis highly likely to develop hypoplastic heart syndrome, pulmonary atresia with intact ventricular septum, pulmonary stenosis and atrial septal interventions are the most widely performed FCIs (59). Alongside with its role in patient selections, echocardiographic guidance is mandatory during the procedures to:

- measure the aortic annulus (60) or the tricuspid annulus and the RV dimension (61), according to the different procedures.
- assist the percutaneous puncture of the maternal abdomen, fetal chest wall and myocardial wall.
- guide atrial septostomy or interatrial stent placement to decompress the left atrium in hypoplastic heart syndrome in patients with intact or restrictive atrial septum (59).

## 3D Imaging and Multimodal Fusion Techniques

The growing diversity and complexity of percutaneous cardiac interventions necessitates true 3D visualization of cardiac anatomy to facilitate optimal planning and tailored treatment. Whilst fluoroscopy remain the cornerstone of imaging in the catheterization laboratory, 3D imaging and multimodal fusion techniques using rotational angiography (RA), CT, magnetic resonance imaging (MRI), and TEE have gained an increasing importance in the interventional workflow. Advanced computing technologies and user-friendly interfaces allows efficient generation of high-quality 3D reconstructions from pre procedural or real time volumetric data sets to plan and guide interventions.

### 3D Rotational Angiography

Rotational angiography uses fluoroscopy C-arm rotation in concert with timed contrast injection to generate multiple 2D datasets that can be reconstructed into high resolution 3D models. Both the dynamic 2D rotational angiogram and 3D reconstruction provide valuable spatial information of the target lesion and relationships to adjacent anatomy such as the airways which is not available from biplane 2D angiography. Several studies have reported an additive diagnostic yield of 3D RA when applied in the evaluation of pulmonary vasculature following cavopulmonary connection in single ventricle patients, which led to changes in patient management (62–64). Because the reconstructed 3D model is in geometric correspondence with the C-arm coordinate system, it can be manipulated to define optimal projections for intervention and overlaid on live fluoroscopy for continuous procedural guidance. These functionalities have been shown to impact positively on the performance and outcomes of various congenital and structural interventions (65–73).

Application of 3D-RA during percutaneous pulmonary valve implantation (PPVI) enables complete evaluation of RV outflow tract morphology and calcification patterns and assessment of potential coronary or aortic compression during balloon inflation at the intended site of valve deployment (70). Further, real time acquisition of RA after positioning of stiff guidewire provides 3D roadmaps with minimal anatomic shift, facilitating precise deployment of the transcatheter valve.

An initial concern with 3DRA is the potential for additional radiation burden. However, published experience of 3DRA for various cardiac interventions have reported comparable or lower exposure rates to standard biplane angiographic acquisitions, particularly following dose optimization strategies (65, 67, 68, 74).

### Image Fusion

Fusion imaging is the overlay of 3D rendered volumes acquired from different imaging modalities on live fluoroscopy for procedural guidance. A pre-requisite for image fusion is the spatial and temporal alignment of images, a process termed image registration.

#### Static Computed Tomography/Magnetic Resonance Imaging-Fluoroscopy Fusion

**FIGURE 5:**
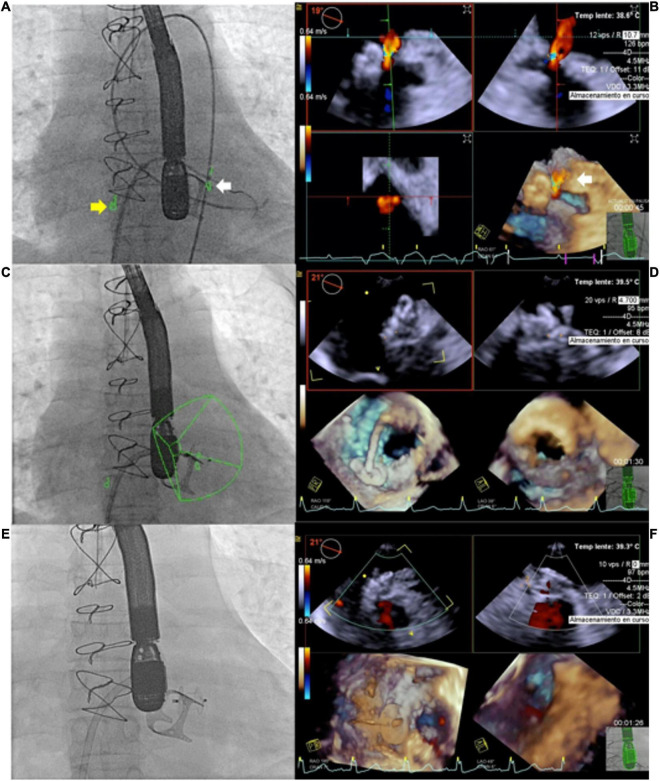
Use of echocardiography-fluroroscopy fusion (EFF) to facilitate mitral paravalvular leak (PVL) closure. **(A,B)** Anatomical markers placed on fluoroscopy and echocardiography. Marker 1 (yellow arrow) indicate position of transeptal puncture. Marker 2 (white arrow) indicate site of mitral PVL. **(B)** Color flow Doppler confirming mitral PVL. Combined EFF image allows efficient crossing of PVL **(C,D)**. Continuous feedback from EFF facilitates alignment of delivery sheath and device deployment. **(E)** Fluoroscopy showed good device position post release, **(F)** confirmed by echocardiography and no residual leak was observed. *Courtesy of Dr. Juan Pablo Sandoval, Instituto Nacional de Cardiologia Ignacio Chavez, Mexico City, Mexico*.

Computed tomography (CT) is the most commonly used modality as the high-resolution acquisition of vasculature and airway allow for easy segmentation and registration. MR imaging has the advantage of avoiding additional radiation exposure and the ability to incorporate cardiac and respiratory motion during acquisition may improve image registration. Multiple approaches have been described for image registration, including automated 2D-3D registration in combination with fiducial markers, or manual refinement with spine, airways, catheter movement or cardiac silhouette as reference points (69, 75–78). Fusion of preprocedural CT/MRI for various interventions such as PPVI (79), coarctation stenting (77), pulmonary artery and vein interventions (69, 75) and paravalvular leak closure [(75), Figure 5], have been reported to improve procedural efficiency, thereby reducing contrast administration and radiation dose (76). Current limitations to image fusion with pre-procedural dataset include registration error caused by differences in patient positioning or motion and distortion of anatomy by rigid wires or delivery sheaths during the procedure. Development of new algorithms and refinement of image registration processes are expected to address these limitations.

#### Dynamic Transesophageal Echocardiography -Fluoroscopy Fusion

Echocardiography- fluoroscopy fusion (EFF) overlays dynamic 2D/3D soft tissue information of the cardiac structure of interest on live 2D fluoroscopy. Distinct advantages of this fusion modality include the ability to show real time changes in anatomy during the procedure, monitor for complications, and comprehensive assessment of post-procedure outcomes, providing important feedback to the operator. Image registration is performed by tracking the movement of the transesophageal probe on fluoroscopy. The EFF system automatically match and align a computer-based model of the TEE probe with the fluoroscopic image of the probe, allowing continuous and instantaneous spatial registration of the two modalities each time fluoroscopy is activated. After registration, the 3D TEE volume (field of view cone) is displayed in the same anatomic alignment as the fluoroscopic projection. Anatomical ‘markers’ can be placed to label the structure of interest. The markers are visible in all views of the 3D TEE volume data and in spatially correlated locations on the fluoroscopic image. Other features including color overlay and 3D anatomic rendering additional valuable information to guide specific interventions. Such exposition of the target lesion/cardiac structure facilitates precise catheter manipulation for crossing of defects or positioning of devices and has been proven useful for ASD and VSD closure, specifically in fenestrated defects (79, 80), percutaneous pulmonary and tricuspid valve implant (79, 81), transeptal puncture and Fontan fenestration creation (69, 82), mitral valve procedures (83, 84) and paravalvular leak closures (82). Several centers have reported decreased fluoroscopy time and radiation dose when using EFF for ASD closure (79). In comparison to image fusion with high resolution CT/MRI, inherent drawbacks of 3D TEE fusion include the limited field of view especially with imaging of anterior structures, variable 3D image resolution, and susceptibility to interference from interventional equipment. Currently, the use of EFF is restricted to patients weighing > 20kgs because the adult 3D TEE probe cannot be placed in small patients (82).

### 3D Printing, Digital Reality Technologies and Holography

**FIGURE 6:**
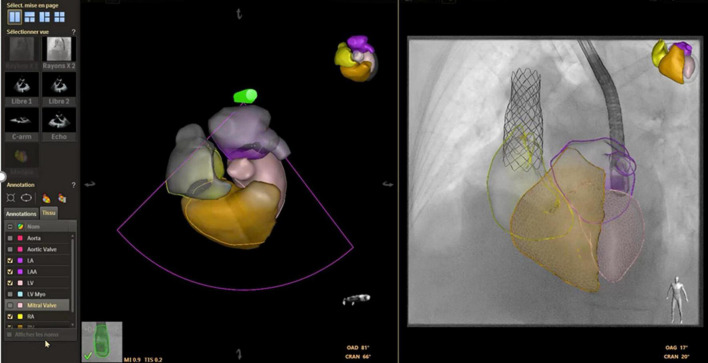
Preprocedural simulation of sinus venosus atrial septal defect percutaneous closure. *Courtesy of Philips Healthcare*.

Despite the advances in 3D imaging techniques, display of 3D data on conventional 2D screen hampers depth perception and appreciation of the complex spatial relations of cardiac structure. Advances in biomedical engineering have led to three-dimensional printing or additive manufacturing that bridges this gap through creation of a physical replica from CT and 3D RA data. The printed models allow true 3D visualization of anatomy (85), procedural simulation (86) and device testing (87), specifically for complex anatomies (88) or high-risk procedures and novel interventional procedures such as the recently described covered stent correction of sinus venosus ASD (89, 90) (Figure 6). Limitations of 3D printed models for procedural simulation are the static representation of the anatomy and structural properties of the printed material that do not respond to implants, ballooning or stenting in the same way as native tissue. Additionally, the time-consuming post-processing and manufacturing required for 3D printing remain important barriers to widespread adoption. Special efforts are being made to integrate physicians’ and biomedical engineers’ work, and this is likely to improve in the next future not only the materials used to build the models but also the quality of the models, making them more realistic and useful (91).

**FIGURE 7:**
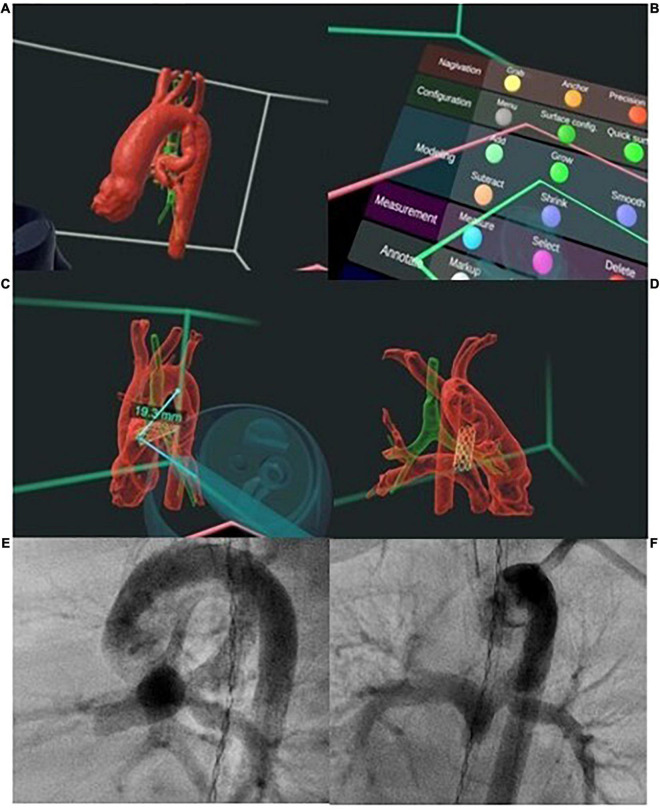
Digital-reality technologies enable true three-dimensional (3D) modeling and simulation prior to patent ductus arteriosus (PDA) stenting. **(A)** The 3D model of the PDA in relation to the airway, can be interrogated **(B)** using a range of interactive features including **(C)** measurement of distances in 3D space and **(C,D)** overlaying of stent in the PDA. **(E,F)** Visualization of the morphology of the 3D model in unlimited planes allows selection of optimal angiographic projection for intervention and planning of the number of stents and length of stent to cover the entire PDA. Post procedure angiography showing position of PDA stent as planned with unobstructed flow into branch pulmonary arteries.

Digital reality technologies have emerged as an alternative 3D visualization platform which can be used not only for planning, but also to assist execution of procedures (Figure 7). The ability for fully automated and instantaneous generation of 3D images from CT/MRI data increases clinical accessibility and utilization. Applications of fully immersive virtual reality technology have been largely confined to pre-procedural planning or education (92, 93). Mixed reality platforms, which allow simultaneous interaction with the virtual and real environment, are intuitively advantageous in the interventional laboratory. Several centers have reported successful use of mixed reality or holographic technologies for instantaneous 3D visualization of imaging data to guide interventions in the cardiac catheterization laboratory (94, 95).

More recently, patient-specific computational models generated from 3D imaging have been developed for virtual planning of intervention and prediction of procedural outcome in pulmonary valve implantation and coarctation stenting. Using simulation methodologies such as finite-element and computational fluid dynamics, deformation of cardiac tissues and blood flow pattern in the heart can be modeled during virtual deployment of devices within the reconstructed surface anatomies (96–98). For each intervention, computational 3D modeling of different device sizes and configuration in the target lesion allows assessment of hemodynamic outcome and anticipation of potential adverse events, which can minimize complications or alter management decisions (98, 99).

## Conclusion

In tandem with the growing diversity and complexity of percutaneous cardiac interventions, imaging must provide reliable visualization of cardiac anatomy to facilitate optimal planning and tailored treatment. 3D echocardiography and multimodality imaging can no longer be considered only as a luxury, but a daily clinical tool, playing a major role on today’s practice and the future to come.

## Data Availability Statement

The original contributions presented in the study are included in the article/supplementary material, further inquiries can be directed to the corresponding author.

## Author Contributions

MA, S-LK, and XI: design and drafting of the manuscript. ZJ: study supervision. XI and J-BT: critical revision of the manuscript for important intellectual content. All authors contributed to the article and approved the submitted version.

## Conflict of Interest

The authors declare that the research was conducted in the absence of any commercial or financial relationships that could be construed as a potential conflict of interest.

## Publisher’s Note

All claims expressed in this article are solely those of the authors and do not necessarily represent those of their affiliated organizations, or those of the publisher, the editors and the reviewers. Any product that may be evaluated in this article, or claim that may be made by its manufacturer, is not guaranteed or endorsed by the publisher.
